# *In Vivo* Imaging and Quantification of Carbonic Anhydrase IX Expression as an Endogenous Biomarker of Tumor Hypoxia

**DOI:** 10.1371/journal.pone.0050860

**Published:** 2012-11-30

**Authors:** Bagna Bao, Kevin Groves, Jun Zhang, Emma Handy, Paul Kennedy, Garry Cuneo, Claudiu T. Supuran, Wael Yared, Milind Rajopadhye, Jeffrey D. Peterson

**Affiliations:** 1 Life Sciences & Technology, PerkinElmer, Inc., Boston, Massachusetts, United States of America; 2 Department of Chemistry, Laboratory of Bioinorganic Chemistry, Università degli Studi di Firenze, Florence, Italy; Seoul National University, Republic of Korea

## Abstract

Carbonic anhydrase IX (CA IX) is a transmembrane protein that has been shown to be greatly upregulated under conditions of hypoxia in many tumor cell lines. Tumor hypoxia is associated with impaired efficacy of cancer therapies making CA IX a valuable target for preclinical and diagnostic imaging. We have developed a quantitative in vivo optical imaging method for detection of CA IX as a marker of tumor hypoxia based on a near-infrared (NIR) fluorescent derivative of the CA IX inhibitor acetazolamide (AZ). The agent (HS680) showed single digit nanomolar inhibition of CA IX as well as selectivity over other CA isoforms and demonstrated up to 25-fold upregulation of fluorescent CA IX signal in hypoxic versus normoxic cells, which could be blocked by 60%–70% with unlabeled AZ. CA IX negative cell lines (HCT-116 and MDA-MB-231), as well as a non-binding control agent on CA IX positive cells, showed low fluorescent signal under both conditions. In vivo FMT imaging showed tumor accumulation and excellent tumor definition from 6–24 hours. In vivo selectivity was confirmed by pretreatment of the mice with unlabeled AZ resulting in >65% signal inhibition. HS680 tumor signal was further upregulated >2X in tumors by maintaining tumor-bearing mice in a low oxygen (8%) atmosphere. Importantly, intravenously injected HS680 signal was co-localized specifically with both CA IX antibody and pimonidazole (Pimo), and was located away from non-hypoxic regions indicated by a Hoechst stain. Thus, we have established a spatial correlation of fluorescence signal obtained by non-invasive, tomographic imaging of HS680 with regions of hypoxia and CA IX expression. These results illustrate the potential of HS680 and combined with FMT imaging to non-invasively quantify CA IX expression as a hypoxia biomarker, crucial to the study of the underlying biology of hypoxic tumors and the development and monitoring of novel anti-cancer therapies.

## Introduction

Carbonic anhydrase IX (CA IX) is a transmembrane cell surface enzyme which catalyzes the reversible interconversion of CO_2_ to bicarbonate and a proton. CA IX is overexpressed in response to tumor hypoxia in many common tumor types [1]–[4] and plays a critical role in hypoxia associated tumor acidosis [5]–[8]. Tumor hypoxia, a result of rapid cell proliferation combined with disordered vascular development [9], [10] and subsequent expression of CA IX, is also correlated to metastasis, poor prognosis and resistance to therapeutic intervention making CA IX an important biomarker in the study of hypoxia, tumor cell proliferation and therapy [11]–[19]. This correlation has led to significant interest in the development of various detection methods for tumor hypoxia and CA IX expression in pre-clinical research and in patients.

Several invasive and non-invasive approaches have been developed to measure tumor oxygenation including the use of oxygen-sensitive electrodes [11], [20], [21] and hypoxia bio-marker targeted agents or antibodies with labels that can be detected by imaging techniques such as positron emission tomography (PET), single photon emission computed tomography (SPECT), magnetic resonance imaging (MRI), autoradiography, and immunohistochemistry [18], [22]–[30]. There has been considerable interest in fluorescent optical reporters for CA IX expression resulting in a range of bodipy-, fluorescein-, and rhodamine-labeled CA IX inhibitors reported for preclinical applications [26], [28], [31]–[36]. In particular, several fluorescein-labeled sulfonamides have been reported for the detection of CA IX in vitro and in superficial tumors in vivo [26], [31]–[33], [37], [38]. However, while near-infrared (NIR) labeled CA IX antibodies for detection of CA IX in breast cancer have been reported [36], [39], none of these fluorescent inhibitors are in the NIR range, which would allow efficient penetration of photons through living tissue and minimize interference from tissue autofluorescence, necessary for deep tissue imaging and optical tomographic quantification [40]–[43].

We have recently reported the synthesis and preliminary evaluation of a series of CA IX-targeted agents employing sulfonamide targeting groups and NIR fluorochromes [44]. The goal of the current studies was to evaluate one of these new agents (HS680) for detecting up-regulation of CA IX by oxygen modulation both in vitro and in vivo and to validate the specific accumulation of the agent in hypoxic regions of CA IX expressing tumors. In vitro activity and selectivity of the agent were tested in four tumor cell lines, CA IX positive HT-29 and HeLa cells and CA IX negative HCT-116 and MDA-MB-231 cells [4], followed by in vivo imaging and quantification in tumor bearing mice by FMT. In vivo FMT imaging results were validated by correlation with ex vivo studies of tumor fluorescence as well as by co-localization with a CA IX antibody, the hypoxia bio-marker pimonidazole and the perfusion stain Hoechst. We report that HS680 has high in vitro and in vivo specificity for CA IX, accumulates preferentially in hypoxic regions of CA IX positive tumors and can be used to non-invasively detect and quantify CA IX in tumors as well as changes in CA IX expression induced by manipulation of oxygen levels.

## Materials and Methods

### Ethical Statement

All in vivo studies were performed in accordance with the recommendations in the Guide for the Care and Use of Laboratory Animals of the National Institutes of Health. The protocol was approved by Institutional Animal Care and Use Committee (IACUC) of the PerkinElmer, Inc. (Protocol Number 01-0904). No invasive or surgical procedures were used in these studies, but all imaging activities were performed under appropriate anesthesia to minimize animal distress.

### Cell Culture

Human cervical carcinoma HeLa (CCL-2), HT-29 (HTB-38), HCT-116 (CCL-247), and MDA-MB-231 (HTB-26) cells obtained from American Type Culture Collection (ATCC, Manassas, VA), were routinely cultured in the ATCC recommended culture medium with 10% fetal bovine serum and 1% penicillin-streptomycin in 75 cm^2^ flasks. Exponentially growing cells between passages 1–10 were used for all experiments. Cells were incubated at 37°C in a humidified atmosphere containing 5% CO_2_. Initial seeding densities of cells were 28,000 to 100,000 cells/cm^2^ (0.1 to 0.3×10^6^ cells/mL) and the volumes of cultures were 0.5 mL and 3 mL, respectively, for 8-well chamber slides and 6-well culture plates. The seeded cells were cultured for 24 hours before hypoxia induction.

### Induction of Cellular Hypoxia *in Vitro*

To induce cellular hypoxia, cell culture plates or slide chambers were placed into a Modular Incubator Chamber (MIC-101, Billups-Rothenberg, Inc. CA) that was infused with mixed-low oxygen gas (1.0% O_2_, 5.0% CO_2_, 94% N_2_) and sealed as instructed by the manufacturer. The sealed modulator chamber was then placed into a CO_2_ incubator for 24 hours along with normoxic culture plates that were directly placed into the CO_2_ incubator with exposure to normal oxygen levels. Cells were evaluated for morphology, increased CA IX expression and pimonidazole binding, and reduced extracellular pH during the evaluation of HS680 for confirmation of hypoxia induction. In all studies, care was taken to ensure that cultures grown in normoxia and hypoxia conditions were subconfluent and contained similar cell numbers. The detailed results and descriptions of materials and methods for validations of cellular hypoxia are presented in (Protocol S1).

### Fluorescence Microscopy of HS680 in Cells

Hypoxic and normoxic cells were incubated with HS680 or non-binding control agent (1 µM final concentration/per well) during the last hour of culture. Allophycocyanin-conjugated anti-human CA IX monoclonal antibody (1∶50 diluted or 100 ng/well, FAB2188A, R&D System) was used as the positive control (Figure S1C). The test agents and antibody were added directly to the culture media (not replaced) to prevent re-oxygenation of the hypoxic cells. Upon completion of 1 hour incubation, the media was discarded and cell wells were rinsed twice with cold PBS and removed from the slides. The slides were dried at room temperature for 4 to 5 minutes, and the nuclear staining reagent DAPI (Invitrogen) was added prior to mounting with a coverslip. The slides were examined under fluorescence microscopy at appropriate fluorescence wavelengths, and images were acquired using an equal exposure time and the same magnification for comparisons.

For blocking studies, the normoxic and hypoxic cells were pre-incubated with 100 µM acetazolamide (AZ) for 1 hour prior to the addition of agents for additional 0.5 to 1 hour in culture. The fluorescence of the cells was visualized by fluorescence microcopy. Each experiment was repeated two to three times with CA IX antibody binding as positive control and with a negative control agent to ensure the validity of assay performed.

### Flow Cytometry and K_d_ Determination

Cells cultured in 6-well plates that were exposed to normoxia and hypoxia, as described above, were incubated with 1 µM HS680 or the control compound during the last 0.5 to 1 hour of the culture. Fluorescein-conjugated mouse monoclonal anti-human CA IX antibody (1∶100 dilution; FAB2188F, R&D System) was used as positive control for flow cytometry assessment (Figure S1C). After collection of cell culture media, cell wells were rinsed with cold PBS and were scraped from the plates using a cell scraper with 2 mL of PBS. Cells were then transferred to 5 mL tubes and spun for 10 minutes at 1000 rpm. To each tube, 500 uL PBS was added to re-suspend the cells for flow cytometry. Blocking of HS680 binding to hypoxic cells was assessed by preincubation of the cells for 1 hour with an excess (100 µM) of AZ.

The dissociation constant (K_d_) of HS680 was assessed by incubating hypoxic and normoxic HeLa cells with increasing concentrations of HS680 for 30 minutes. The cells were then prepared for flow cytometry as described above to determine mean fluorescence (binding). The K_d_ value of HS680 was calculated using GraphPad Prism 5 Curve Fitting Software with binding values to normoxic cells considered as non-specific binding and binding values to hypoxic cells defined as total binding.

### Tumor Xenograft Models and *in Vivo* Hypoxia Induction

For in vivo studies, female *nu/nu* mice at 4–5 weeks old, obtained from Harlan Laboratory (Indianapolis, IN), were injected subcutaneously (s.c.) with HT-29, HeLa, HCT-116 or MDA-MB-231 cells (1.5×10^6^ cells/site) at the mammary fat pads or flank region. Once tumors reached the desired volume of 600–700 mm^3^ (measured with calipers using the formula: volume mm^3^ = length×width^2^/2), mice were grouped randomly (n = 4–5 mice/group) for in vivo experiments. Xenograft tumors with the volume of 600–700 mm^3^ are known to be hypoxic [45]–[47]. HS680 or control agent (2–4 nmol formulated in 1X PBS was injected intravenously and mice were imaged by tomographic imaging on the FMT at the appropriate time point(s). The administered doses of imaging agents were far below the therapeutic dose of AZ and therefore agent toxicity was not expected. To demonstrate in vivo blocking of HS680 signal, the mice were injected with AZ (10 mg/kg, iv) 1 hour before injection of HS680. In some studies, mice bearing HeLa xenografts (300–400 mm^3^ in volume) were exposed to controlled hypoxic environmental conditions (8% oxygen). To do this, mice were kept for 48 hours in a custom-made, sealed-environmental chamber with gas infusion (bottom left side) and escape (top right side) adaptors. Oxygen levels within the chamber were monitored regularly to make sure that 8% oxygen was maintained for the duration of the study. Control mice with matched tumor volumes were maintained under normal housing conditions. HS680 (2 nmol) was injected intravenously to all mice at 24 hours after the initiation of the experiment. Hypoxic mice were placed back in the hypoxic chamber after the HS680 injection, and tomographic imaging was performed on the FMT 24 hours following injection.

### *In Vivo* FMT and Measurements

Tumor-bearing mice were anesthetized by isoflurane/oxygen mixture gas anesthesia system. Experimental and control mice were then imaged using fluorescence tomography (FMT 2500^LX^ Pre-Clinical Imaging System, PerkinElmer, Boston, MA PerkinElmer, Boston, MA). Briefly, the anesthetized mice were positioned in the imaging cassette which was then placed into the FMT imaging chamber. A NIR laser diode transilluminated (*i.e.* passed light through the body of the animal to be collected on the opposite side) the tumor regions, with signal detection occurring via a thermoelectrically cooled CCD camera placed on the opposite side of the imaged animal. Appropriate optical filters allowed collection of both fluorescence and excitation datasets, the entire image acquisition sequence taking approximately 5–6 min per mouse. The collected fluorescence data was reconstructed (TrueQuant™ software, PerkinElmer, Boston, MA) for the quantification of three-dimensional fluorescence signal within the tumors. Three-dimensional regions of interest (ROI) were drawn in the upper torso encompassing each tumor region. A threshold was applied identically to all animals (equal to 30% of the mean tumor fluorescence of positive control mice). For visualization and analysis purposes, TrueQuant software provided three dimensional (3D) images and quantification in pmoles of fluorescence within ROIs. Additional technical information on FMT imaging, image re-contruction, image analysis and quantification can be found in the following review articles [48], [49].

### Pharmacokinetics of HS680

Twenty seven CD-1 female mice (retired breeder with 35–40 g body weight) were injected intravenously with 2 nmol HS680. Terminal blood samples were collected at nine time points by cardiac puncture from each mouse following carbon dioxide asphyxiation. Approximately 500 µL of whole blood were collected from each study animal at pre-dose, 0.016, 0.083, 0.5, 1.0, 2.0, 4.0, 6.0, and 24 hours post-injection (n = 3 mice/time point). Blood samples were collected into tubes containing dipotassium EDTA (K_2_ EDTA) as the anticoagulant. For each sample, the tube was inverted approximately 5 times immediately to ensure effective anticoagulation. Samples were initially placed on wet ice until processed into plasma. The tubes containing blood were centrifuged at 14,000 rpm for 10 min at 4°C and the plasma samples were collected into a new set of tubes and stored at −80°C freezer until analyzed. To determine the fluorescence intensity and concentrations of HS680 within the plasma samples, 100 uL of each plasma sample and standards were transferred to a 96 well plate and the fluorescence intensity was read using a Gemini-XS plate reader (Molecular Devices, Sunnyvale, CA). In order to determine the concentrations of HS680 within the plasma samples, a standard curve was created using HS680 at various concentrations (2.2, 0.4 0.08, 0.04, 0.009, 0.004, and 0.0009 µM). The half-life of HS680 within plasma was analyzed using Graphpad Prism 5 Software using a two phase decay method.

### *Ex-vivo* Fluorescence Reflectance Imaging (FRI) and Measurements

After completion of in vivo non-invasive 3D tomographic imaging on the FMT as described above, mice bearing tumors were sacrificed by carbon dioxide asphyxiation and tumors or organs were excised and placed on imaging blocks within the imaging chamber as a group, and imaged on FMT2500LX using the two dimensional (2D) reflectance mode (ex-vivo FRI 2D imaging). Using TrueQuant Software, regions of interest (ROIs) were drawn around each tumor or organs and the mean fluorescence signal was determined, analyzed, and reported as Mean±Standard Error of the Mean (SEM) in relative fluorescence units (rfu) of counts/energy (signal intensity normalized to the power of the light source). Correlation analysis of 3D tomographic and 2D reflectance imaging was done for some experiments to confirm that signals measured in vivo correspond to signals determined ex-vivo.

### Immunohistochemistry and Fluorescence Microscopy of HS680 in Tumors Tissues

After FMT imaging of tumor bearing mice injected with HS680 or the control agent, mice were then injected intravenously with pimonidazole (80 mg/kg) and Hoechst 33342 (25 mg/kg) one hour and five minutes before sacrifice, respectively [45], [46]. Tumors, kidney and muscle tissues were collected and imaged by FRI. The tissues were then embedded, frozen in OCT, and stored at −80°C until they were sectioned for fluorescence microscopy and immunostaining. Sections of 8 µm thickness were prepared and air dried for 10 min. Sections were imaged for Hoechst (blue) in combination with the control agent or HS680 (red). After acquiring images, the sections were fixed in ice-cold acetone for 20 min and incubated in SuperBlock (37515, Thermo Scientific) for 30 min. The tissue sections were then stained with detection antibodies diluted with blocking solution for 1 hour; CA IX expression was detected with fluorescein-conjugated Anti-Human CA IX monoclonal antibody (FAB2188F, R&D System) diluted 1∶10 and pimonidazole binding was detected by FITC-conjugated murine anti-pimonidazole monoclonal antibody (provided in the Hypoxyprobe plus kit) diluted 1∶25 to a adjacent section. The sections were imaged again in blue (Hoechst) and green (pimonidazole and CA IX) fluorescence. Finally, the sections were stained with H&E according to standard protocol. Images were taken by Carl Zeiss Axiovert fluorescence microscope. All images were taken at 25× magnification (2.5× objective) with identical exposure times.

### Statistical Analysis

All pairwise comparisons were analyzed using a two tailed student’s *t*-test in Excel. Most of the data presented as Mean±SEM. All error bars represent the SEM. A *p* value equal to or less than 0.05 was considered significant.

## Results

### *In Vitro* Characterization of HS680

**Figure 1:**
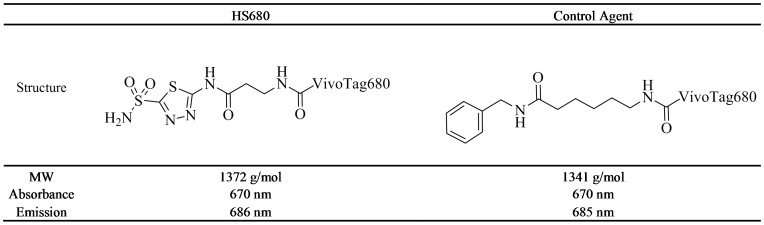
Structures, molecular weight (MW) and maximum optical absorption and emission wavelengths (1×PBS) for HS680 and the control agent.

**Figure 2:**
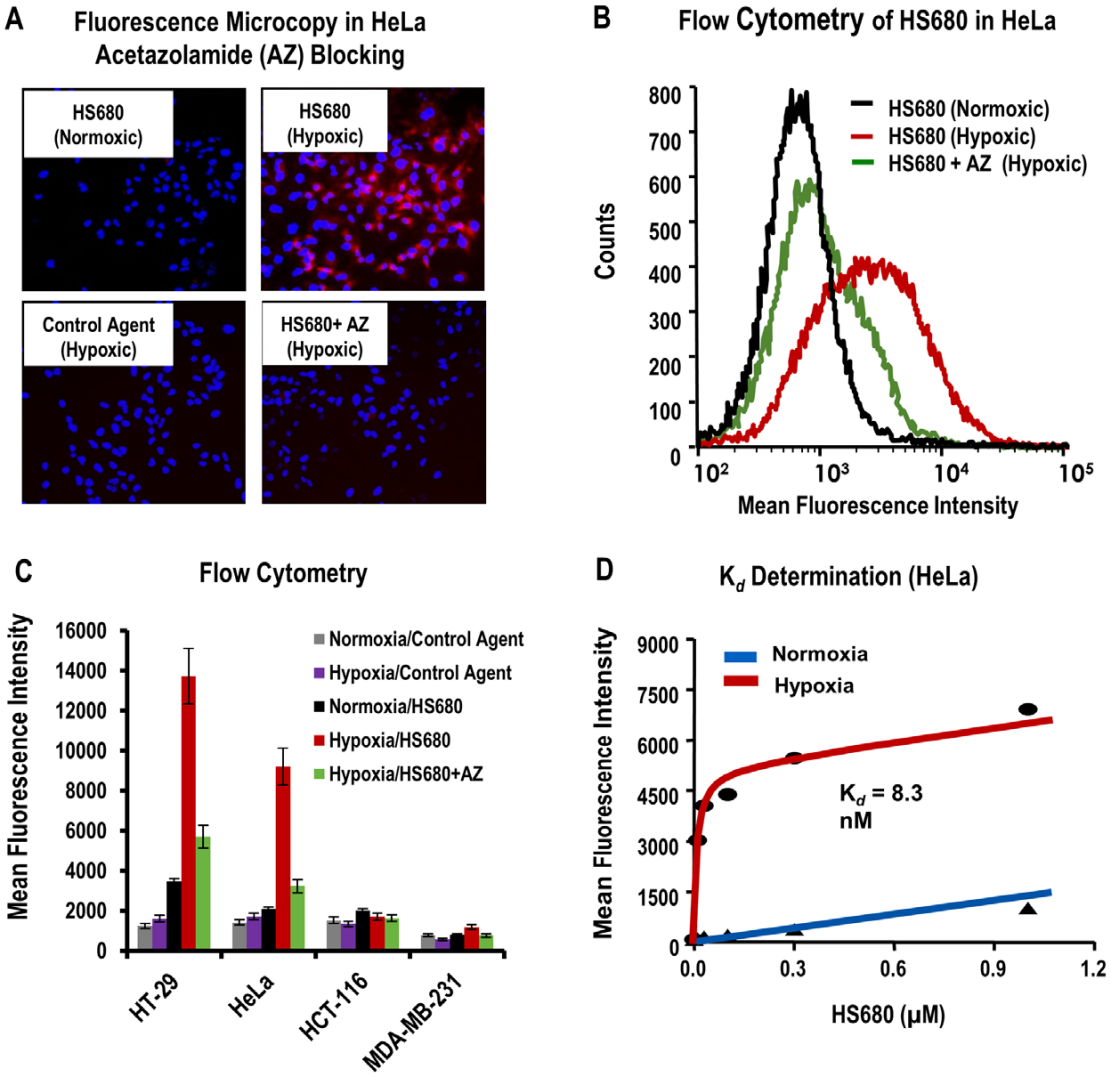
In vitro characterization of HS680 in hypoxic and normoxic tumor cells. A, Binding of HS680 to hypoxic and normoxic HeLa cells was shown with fluorescence microcopy. Cells labeled with the control agent cultured under hypoxic conditions provided a negative control. Blocking of HS680 binding by pre-incubating the hypoxic cells with AZ is shown in lower right panel. The images of cells cultured with control agent and HS680 with AZ blocking under normoxic conditions were similar to the images of HS680 cultured at nomoxic conditions, and therefore they were not shown. B, Representative histograms of HS680 binding to normoxic and hypoxic HeLa cells by flow cytometry with AZ blocking in hypoxic cells. C, Flow cytometry quantifications of binding of HS680 and control agent to normoxic and hypoxic HT-29, HeLa, HCT-116, and MDA-MB-231 cells with or without AZ blocking in hypoxic cells. D, The disassociation constant (K_d_) of HS680 was determined in HeLa cells by flow cytometry to be 8.3 nM.

The synthesis and characterization of HS680 and the control agent used in these studies was reported previously [44]. The structures, physical properties and CA inhibition profiles are shown in Figure 1 and Table 1, respectively. HS680 shows increased inhibition of CA IX relative to the parent inhibitor (7.5 nM vs 25 nM acetazolamide, AZ) in addition to improved selectivity over CA II, XII and XIV [44]. To visualize and quantify binding of HS680 to CA IX positive cells, HT-29 and HeLa cells were incubated with the agent or the control under normoxic and hypoxic conditions and imaged by fluorescence microscopy (Figure 2A) or quantified by flow cytometry (Figure 2B and 2C). HS680 effectively detected upregulation of CA IX in these cells under hypoxia relative to normoxia, whereas the non-binding control agent did not label the cells under either condition, and was comparable to HS680 in normoxic cells. Cells pre-incubated with an excess of the parent CA IX-inhibitor AZ blocked the signal in hypoxic cells by 60%–70% (Figure 2A, 2B, and 2C). Assessments of HS680 binding to the CA IX negative cell lines HCT-116 and MDA-MB-231 showed low signal and comparable to the nonbinding control agent under both normoxic and hypoxic conditions (Figure 2C). The induction of cell hypoxia in the hypoxic chamber and subsequent expression of CA IX relative to normoxia was validated in HT-29 and HeLa cells using pimonidazole staining and binding (Figure S1A), measurement of CA IX protein levels by CA IX ELISA of HT-29 anf HeLa cell lysates (Figure S1B) or by flow cytometry of anti-CA IX antibody binding to four different cell lines (Figure S1C), and detection of a decrease in media pH (Figure S1A). All measurements were consistent with cellular hypoxia, and increased CA IX levels were detected for hypoxic HT-29 and HeLa cells (CA IX positive). As expected, CA IX upregulation was not detected in CA IX negative HCT-116 and MDA-MB-231 cells under hypoxic cultures (Figure S1A, S1B, and S1C). These results confirmed that HS680 bind to hypoxic cells expressing CA IX that can be detected and quantified with anti-CA IX antibody binding assays.

**Table 1:** Inhibition data for CA II, IX, XII and XIV for the parent CA inhibitor AZ, HS680 and the control agent as determined by stopped-flow CO_2_ hydration method.

	K_i_ (nM)				K_i_ Ratios		
Compound	hCA II	hCA IX	hCA XII	hCA XIV	hCA II/IX	hCA XII/IX	hCA XIV/IX
**AZ**	12	25	5.7	41	0.5	0.2	1.6
**HS680**	248	7.5	35	66	33.1	4.7	8.8
**Control**	>1000	>1000	>1000	>1000	–	–	–

The binding affinity to hypoxic cells was further assessed by measuring the dissociation constant (K_d_) in hypoxic HeLa cells by flow cytometry (Figure 2D). Hypoxic and normoxic cells were incubated with a range of concentrations of HS680 from 10 nM to 1 µM, and the mean fluorescence of the cells at each concentration was quantified by flow cytometry. Signal from the normoxic cells, which were previously determined to express very low levels of CA IX, was used as a measure of nonspecific uptake. The K_d_ calculated for HS680 was 8.3 nM, a value in excellent agreement with the measured inhibition constant (K_i_ = 7.5 nM). In this assay, it was noted that HS680 provided 25-fold higher signal in hypoxic cells as compared to signal in normoxic cells at low doses close to the K_d_ value, and a 7-fold increase was still observed even at the high, saturated dose of 1 µM (Figure 2D).

### *In Vivo* Imaging of HS680 in Mice Bearing HeLa Tumors

**Figure 3:**
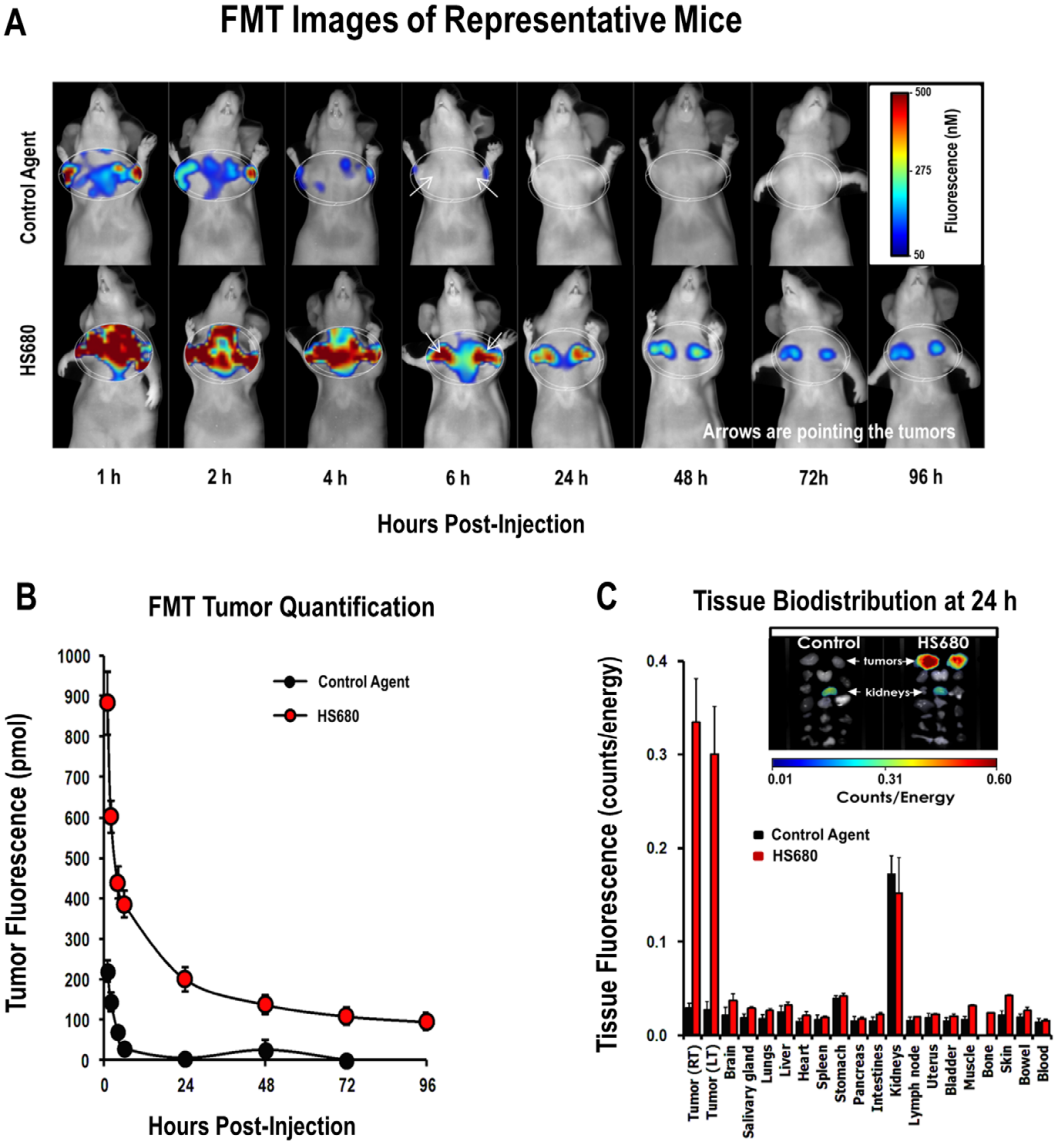
In vivo imaging of HS680 clearance from HeLa tumors and terminal bio-distribution of HS680 in mouse tissues. A, Representative images of tomographic imaging of tumor bearing mice at various times after IV injection of the control agent and HS680, showing progressive changes in tumor fluorescence signals. B, Tumor fluorescence concentrations were quantified by the FMT at various times after the agent injection, showing the highest contrast of HS680 over the control agent background signal at 24 hours post injection. C, Bio-distribution of the control agent and HS680 in mice bearing HeLa tumors was determined from the 2D FRI images of excised organ tissues, removed 24 hours post-agent injection. Representative images of tumors and organs are inset with arrows indicating tumors and kidneys. High mechanistic fluorescence signal was observed only within tumors of HS680 injected mice, and the high kidney fluorescence seen with both agents was attributed to renal clearance.

The in vivo accumulation of HS680 fluorescence in HeLa tumors implanted in mice was imaged and quantified by FMT and compared to the non-binding control molecule, which was used as a measure of non-specific uptake and clearance from tumor and surrounding tissues. Fluorescence tomographic images of mice injected with either the control agent or HS680, and imaged sequentially over a 96 h time period, are shown in Figure 3A (upper and lower panels each following a representative individual animal over time). Both agents showed rapid distribution into tumor tissues as well as surrounding areas and quick washout from non-tumor regions (4 to 6 h). HS680 fluorescent signal was retained in the tumors (quantified in Figure 3B), with significant contrast and tumor definition apparent in the tumor regions as early as 6 hours, while the control molecule had essentially washed out by this time point (Figure 3A and 3B). Supportive plasma pharmacokinetic assessment was performed in parallel in CD-1 mice in the absence of tumor xenografts showing a calculated apparent plasma half-life for HS680 of approximately 2 minutes and near-complete clearance from circulation by 6 hours (Figure S2), consistent with the observed FMT tumor imaging results. Quantitatively, the fluorescence concentrations within the tumors (Figure 3B) showed progressive reduction over time with the highest contrast of HS680 over the control agent background signal observed at 24 hours post injection. Overall, a 10- to 40-fold excess tumor signal in the HS680 injected mice versus control agent injected mice was observed from 6 to 48 hours.

### *Ex Vivo* Imaging of HS680 Organ Distribution in Mice Bearing HeLa Tumors

Assessment of the whole body biodistribution of HS680 in tumor bearing mice provides useful information regarding selectivity of agent localization, route of clearance, and organs/tissues that may show high background signal. Tissues were collected from tumor-bearing mice at an optimal imaging time point (24 hour post injection) and imaged on the FMT 2500 using 2D reflectance mode. Biodistribution results for HS680 and the control agent in HeLa tumor bearing mice are shown in Figure 3C and confirm the selective localization of HS680, but not the control agent, to tumor tissue. HS680 further showed little detectable fluorescence in any other organ except for the kidneys, suggesting predominant kidney clearance. The control agent showed appreciable fluorescence only in the kidneys (Figure 3C). Quantification of the FRI images of the organs, ex-vivo, showed greater than 10-fold excess of fluorescence signal in tumors from HS680 injected mice compared to other organs (except kidney) or tumors from the control agent injection, in agreement with the in vivo results obtained by quantitative tomography.

### *In Vivo* Selectivity of HS680 in Mice Bearing HT-29, HeLa, HCT-116, and MDA-MB-231 Xenografts

**Figure 4:**
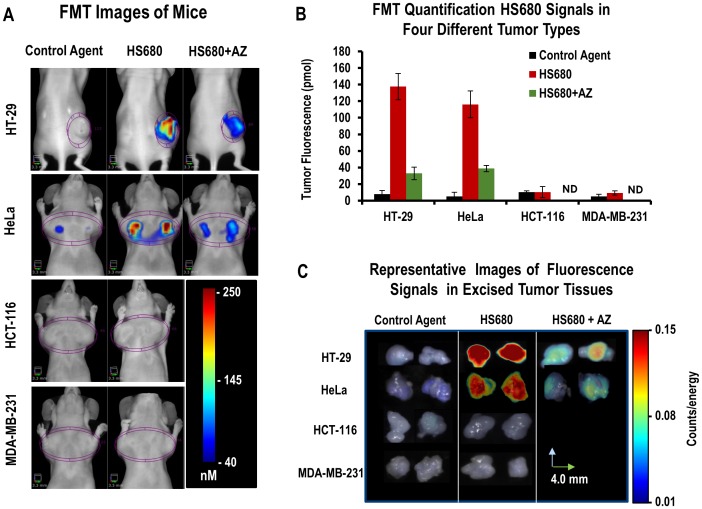
In vivo imaging of HS680 and control agent in mice bearing CA IX positive (HT-29 and HeLa) and CA IX negative (HCT-116 and MDA-MB-231) tumors, with and without AZ competition. A, Representative FMT images and B, Tomographic quantitative analysis of tumor bearing mice injected with control agent and HS680 showing significant accumulations of HS680 fluorescence signals within HT-29 and HeLa tumors, but not in HCT-116 and MDA-MB-231 tumors, and less in the mice that were pre-injected with 10 mg/kg AZ (HS680+AZ) 1 hour before HS680 injection. Mice injected with control agents did not show any appreciable tumor fluorescence signals. HT-29 tumors are at the flank regions of the mice while all other tumor types are implanted in the mammary fat pads, and all tumors are housed with a 3D region of interest (ROI) in each image. *ND* indicates “Not determined.” C, Representative FRI images of four types of tumors collected following the FMT imaging, showing ex-vivo validation of in vivo imaging results.

To further demonstrate the in vivo CA IX specificity of HS680 signal quantified in tumors, we investigated HS680 tumor fluorescence in mice bearing CA IX negative HCT-116 and MBA-MD-231 tumors and compared the results with mice bearing CA IX positive HT-29 and HeLa tumors. Representative mouse images of all four tumor lines are shown in Figure 4A indicating that HS680 signal was observed only in HT-29 and HeLa tumors. Consistent with in vitro flow cytometry results (Figure 2C), quantified HS680 signal was low and comparable to control agent signal in the CA IX negative HCT-116 and MDA-MB-231 tumors (Figure 4A). In contrast, the CA IX positive HeLa and HT-29 tumors accumulated an average of 13X the signal of the negative tumors, as quantified by FMT. The results of FMT in vivo quantification from all four tumor lines are shown in Figure 4B. Furthermore, in vivo blocking, similar to studies performed in vitro, using intravenous pre-treatment of mice with 10 mg/kg AZ resulted in ∼70% knockdown of HS680 in vivo signal (Figure 4A, 4B, and 4C). The ex-vivo 2D tumor fluorescence of from all four lines was in full agreement with the tomographic in vivo quantification (Figure 4C and 4B). AZ blocking of HS680 signal in HCT-116 and MDA-MB-231 tumor bearing mice was not performed because the low levels of HS680 signal was detected in the tumors.

### *In Vivo* Regulation of HS680 Tumor Fluorescence Signal by Oxygen in Mice Bearing HeLa Tumors

**Figure 5:**
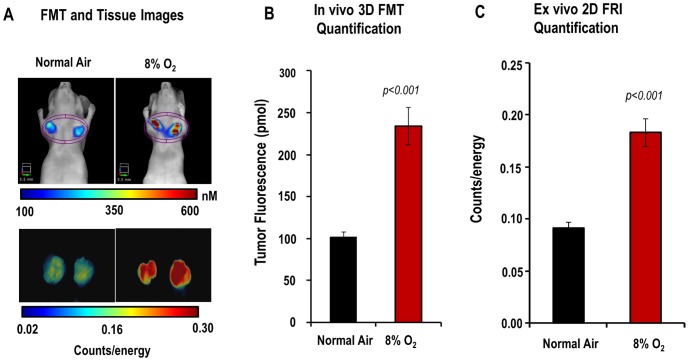
The effect of breathing 8% O_2_ on HS680 tumor signal in HeLa xenografts. Two groups of mice (n = 4 per group) were used to assess the effect of oxygen breathing levels on HS680 labeling of small tumors. A, Representative FMT 3D images of mice breathing normal air *(top left)* and breathing low oxygen *(top right)* showing the intensity differences of HS680 signals within the tumors. FRI 2D images of tumors excised from mice that were placed on normal air (*bottom left*) and 8% O_2_ (*bottom right)* showing again the intensity of fluorescence in tumors. B and C, Quantitative analysis (3D and 2D, respectively) of HS680 tumor signals in mice breathing normal or low oxygen, showing greater tumor HS680 signals in a mice breathing low oxygen than that of mice breathing normal air.

To investigate whether a reduced oxygen environment (hypoxia) for mice bearing HeLa xenografts could further manipulate CA IX expression and HS680 accumulation in tumors, a 48 hour mouse hypoxia experiment was conducted. Representative images and differences in fluorescence signal intensity of mice breathing normal air and low oxygen and the excised tumors from the same mice are shown in Figure 5A. Quantification of HS680 signal accumulation within tumor regions showed a 2.3-fold increase (*p*<0.001) in mice breathing low oxygen as compared to mice breathing atmospheric oxygen (Figure 5B). The intensity of fluorescence measured from ex vivo 2D FRI of tumors excised from both groups of mice confirmed the results of in vivo measurements (Figure 5C). Imaging of non-tumor bearing nude mouse controls showed only background signal within the comparable anatomical region (data not shown).

### Tissue Localization of HS680 in CA IX Positive Tumors

**Figure 6:**
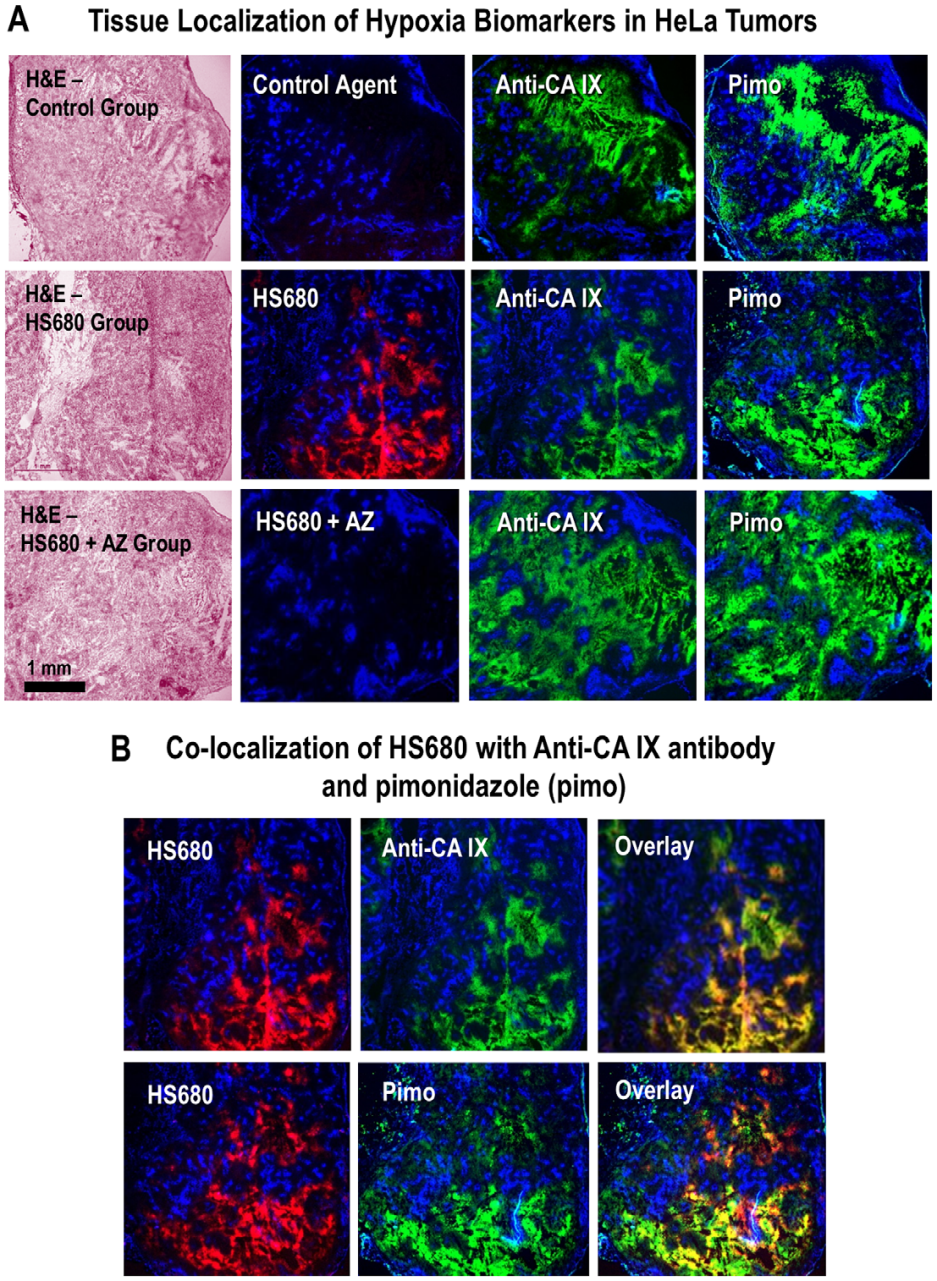
Localization of HS680 and tumor hypoxia markers in HeLa xenografts. A, The tissue staining patterns of the control agent, HS680, and HS680+AZ (red) from the same or adjacent tumor sections as staining with fluorescent CA IX antibody or pimonidazole (green) and the Hoechst perfusion stain (blue). H&E staining of tissue sections that were used for localization are shown on the left side of images. HS680 was specifically localized in regions with low Hoechst staining indicative of low oxygen (less perfused) but positive staining with both the CA IX antibody and Pimonidazole. Preinjection of the mice with unlabeled AZ blocked the binding of HS680 to control levels. B, Co-localization (overlay) of HS680 with CA IX antibody or pimonidazole (hypoxyprobe) is shown on the right side images indicating HS680 was clearly co-localized with both anti-CA IX antibody and hypoxyprobe (pimonidazole). Similar results were obtained using HT-29 tumors (Figure S4A and Figure S4B).

To characterize the distribution of HS680 fluorescence in CA IX expressing tumors, HeLa tissues were collected from mice that received either HS680 or the control agent for assessment by fluorescence microscopy. CA IX expression was visualized with a fluorescent CA IX antibody applied to ex vivo tissue slices, tumor hypoxic regions were visualized by i.v. administration of pimonidazole (HypoxyProbe) followed by ex vivo staining with a fluorescent antibody, and non hypoxic regions were identified by injection of Hoechst stain (a blood perfusion marker) 5 minutes prior to sacrifice, all 24 hours post injection of HS680 or control. HS680, anti-CA IX antibody, and pimonidazole all co-localized to the same tumor regions, while the non-binding control agent did not bind to any regions of the tumors (Figure 6A). Furthermore, the tissue staining patterns of HS680, CA IX antibody and pimonidazole were specifically concentrated in regions with low Hoechst staining, indicative of highly perfused and well oxygenated regions. Overlays of the staining pattern of all three agents (HS680, CA IX antibody and pimonidazole) in HeLa are shown in Figure 6B, showing good co-registration of HS680 labeling (in vivo) with both Anti-CA IX antibody and Anti-pimonidazole antibody staining in tissue sections. Pre-injection of the mice with unlabeled AZ blocked the binding of HS680 to tumor tissues, but, as expected, had no effect on the CA IX antibody or pimonidazole staining (Figure 6A). Immunohistochemistry analysis of skeletal muscle, a tissue frequently used to determine the background signal in radionuclide-based imaging, showed no staining with CA IX antibody, pimonidazole, or HS680. In the kidneys, the observed HS680 signal was mostly distributed in the cortex areas where there was no evident anti-CA IX antibody or pimonidazole staining, supporting our contention that the kidney is merely a route of in vivo clearance of HS680 and the control agent (Figure S3).

## Discussion

A wide range of biological pathways are activated in tumors in response to hypoxia which result in changes in energy metabolism, neovascularization, microenvironment pH regulation and cell migration. [19], [47]. These adaptations facilitate tumor growth and survival as well as conferring resistance to traditional chemo- and radiotherapies. One such pathway is the upregulation in expression of CA IX in response to hypoxia inducible factor 1a (HIF-1a) which, in conjunction with various ion transporters, enables acidification of the extracellular environment with excess protons formed intracellularly from glycolytic metabolism [5], [38]. This pH regulating effect enhances tumor cell survival under hypoxic conditions and contributes to the evolution of metastatic and drug-resistant phenotypes [19], [50].

CA IX is a unique biomarker of hypoxia because it is present in few normal tissues (liver, colon, stomach and heart) yet upregulated to high levels under hypoxia on the cell surface of many tumor types. This makes it an ideal candidate for a targeted fluorescent imaging agent, since it is an endogenous marker that presents an accessible extracellular binding site reflecting the intracellular hypoxic state of the cell. Despite the fact that CA IX is not upregulated in all cancer cell lines [4], a variety of spontaneously arising human tumors, including CNS, head & neck, lung, breast, colon, cervical, ovarian, prostate, renal cancers, leukemia, and melanoma are known to upregulate CA IX expression, making this molecule a clinically relevant biomarker for hypoxic tumors. In addition, a variety of other tumors show increased focal expression of CA IX, occurring in >90% of cervical, glioblastoma, and basal cell carcinomas, as well as in 25–30% of non-invasive and invasive ductal breast cancers with a high correlation with poor histological grade [1].

A number of studies have employed radio- or fluorescent-labeled CA IX antibodies [27], [28], [40], [51]–[53] and CA IX inhibitors [18], [26], [31]–[33], [36] for the detection or characterization of hypoxia-associated CA IX expression. However, the majority of these previous studies relied on ex-vivo evaluation of tumor tissues or limited 2D FRI imaging in vivo. Although NIR-labeled antibodies have been explored [39], none of these previously reported CA IX inhibitor agents took advantage of the benefits of NIR fluorophore-labeling for quality and depth of in vivo imaging. Furthermore, a common limitation of antibody based imaging agents is slow or incomplete tissue distribution, particularly to poorly vascularized regions that are associated with hypoxia [51]. We recently reported CA IX binding and preliminary imaging of a series of NIR-fluorescent CA inhibitors designed to overcome the limitations of previously reported agents [44], and identified a promising candidate, HS680. However, a comprehensive study across multiple tumor cell lines in vitro and in vivo with validation against orthogonal markers of hypoxia has not been reported. In the present study, we aimed to establish HS680 as an in vivo imaging agent for selective and quantitative imaging of CA IX expression non-invasively as a marker of tumor hypoxia.

The specificity of HS680 for cell surface CA IX, particularly over the ubiquitous intracellular CA II, as well as low nonspecific binding to CA IX negative cells, are of key importance for a robust in vivo readout. HS680 employs the well-characterized CA IX inhibitor AZ as the CA IX targeting moiety linked to an NIR fluorescent reporter. A limitation of AZ is specificity among various CA isoforms, however the 33-fold selectivity of HS680 for CA IX over CA II measured by inhibition of CO_2_ hydration is a considerable improvement over the parent inhibitor, which has a modest preference for CA II (Table 1). More importantly, however, the negatively charged NIR dye used to synthesize this conjugate provides low cell permeability [44], which should minimize cell uptake and enhance the selectivity toward membrane bound CAs in a cellular context. Indeed, intracellular accumulation in CA IX negative cells was found to be low for HS680, comparable to the non-CA binding control molecule, bearing the same dye linked to an inert benzylamine moiety. Selectivity over the membrane bound CA XII is somewhat lower, but CA XII has also been identified and targeted as a marker of hypoxia with a lower degree of expression than CA IX [38], [52], so some off target binding to CA XII will likely not interfere with CA IX imaging of hypoxia [38], [52]. To evaluate the agent in vitro, we took a multifaceted approach employing four cell lines determined to become CA IX positive when under hypoxic conditions, or be constitutively CA IX negative. Establishment of a state of cellular hypoxia during incubation and resultant expression of CA IX was confirmed by traditional means (Figure S1). Quantitative flow cytometry, in addition to fluorescence microscopy, was performed on all cell lines under normoxic and hypoxic conditions with HS680, the nonbinding control molecule and with blocking of HS680 binding by unlabeled AZ. Significant binding was observed with CA IX positive HT-29 and HeLa cells when hypoxic, with normoxic signals comparable to signal from the control agent or CA IX negative cells, in agreement with the measured response to a fluorescent CA IX antibody. Blocking of the signal from hypoxic HT-29 and HeLa cells by unlabeled AZ and calculation of a cellular K_d_ comparable to the K_i_ determined with isolated enzyme demonstrate selective and potent targeting of cell surface CA IX expression in vitro, in addition to low non-specific accumulation.

The in vivo results obtained by FMT imaging of HS680 in tumor bearing mice closely matched the in vitro results obtained with each cell line. Once again, only CA IX positive HT-29 and HeLa cells showed significant signal, while CA IX negative cell lines HCT-116 and MDA-MB-231 as well as the control agent in all four cell lines showed minimal tumor accumulation. Maximal contrast was observed around 24 h with very high target to background ratios, but good contrast and tumor definition was observed as early as 6 h post injection. The in vivo signal could also be blocked by AZ, further confirming the in vivo specificity of the agent. Both quantitative in vivo tomography (FMT) and 2D ex vivo FRI imaging of excised tumors gave similar results, and nonspecific background accumulation in non-tumor tissue was limited to the kidneys. While most tumors are expected to have some regions of hypoxia, we attempted to use HS680 to quantify an increase in expression of CA IX by relatively small tumors, which are reported to be less hypoxic [45]–[47], in response to forced hypoxia by maintenance of the mice in a low oxygen (8% O_2_) environment relative to those breathing normal air. The observed >2-fold increase in HS680 signal, and correlation with ex vivo images of excised tumors, confirms that changes in CA IX expression levels as a result of lower oxygen levels can be quantified non-invasively by FMT.

Importantly, HS680 was shown by imaging of ex vivo tissue sections to be specifically localized in hypoxic regions of the tumors. Well perfused and oxygenated regions (*i.e.* non-hypoxic) of HeLa tumors indicated by the Hoechst stain were distinctly different from the regions where HS680, anti-CA IX antibody, and pimonidazole were all colocalized. Skeletal muscle, a tissue frequently used to determine the background signal in radionuclide-based imaging, showed no staining with CA IX antibody, pimonidazole, or HS680. The observed HS680 signal in kidney cortex area where CA IX antibody and pimonidazole staining were negative is consistent with kidney clearance of the agent and was similar to the control agent. Of the four different readouts (HS680, anti-CA IX antibody, pimonidazole, and Hoechst) in tumors, only HS680 can be quantified non-invasively in vivo in addition to ex vivo imaging. These results also indicate the efficacy of HS680 in penetrating even poorly vascularized tumor tissue, a critical property for a non-invasive hypoxia agent.

### Conclusions

We have shown non-invasive, quantitative imaging of CA IX expression in tumor xenografts with an NIR-labeled inhibitor, HS680, and correlated the in vitro and in vivo characteristics of the agent across four different cell lines with varying degrees of CA IX expression. We have demonstrated positive manipulation of the magnitude of tumor signal by restriction of atmospheric oxygen, and we have further demonstrated a spatial correlation between the in vivo signal of HS680 and orthogonal markers of tumor oxygenation by ex vivo microscopy of tumor slices. Taken together, we have validated the use of FMT imaging with a selective, NIR fluorescent CA IX inhibitor for non-invasive quantification of CA IX expression as an indicator of tumor hypoxia in live animals bearing CA IX positive tumor xenografts. HS680 should provide a powerful tool for hypoxia research across the in vitro to in vivo spectrum for pre-clinical and drug discovery applications, and potentially a future tool for translation into clinical interrogation of tumors in patients.

## Supporting Information

Figure S1 **Validation of cellular hypoxia in vitro.** A, Hypoxic induction in HT-29 cells was validated by pimonidazole (Pimo) binding using fluorescence microscopy and flow cytometry and by measuring culture media pH. HT-29 cells showed high levels of pimonidazole (exogenous hypoxia bio-marker) binding when cultured under hypoxic conditions, but not under normoxic conditions, very little signal was observed when the cells were incubated with FITC labeled anti-pimonidazole antibody (no pimonidazole) or with no agent (media) showing that pimonidazole binding was specific to hypoxic cells. The expected acidification associated with hypoxic cell culture conditions was confirmed by measuring the pH of the culture media. B, Quantification of CA IX protein levels in hypoxic and normoxic HT-29 and Hela cell lysates by CA IX ELISA. Effects of cell types and seeding cell densities are shown. CA IX protein was up-regulated in both cell types when cells were cultured in hypoxic condition, increasing 5 to 20 fold depending on the cell densities. An increased expression of CA IX was known when HT-29 cells cultured at higher densities in normoxic cultures. Under hypoxic cultures, CA IX expression in HT-29 cells was further increased 3 to 5 fold. In contrast, HeLa cells expressed very low levels CA IX protein in under normal oxygen conditions, regardless of cell density, with CA IX protein up-regulated greatly (>20-fold) in under low oxygen conditions. Little or no CA IX was detected in the cells prepared for the cultures (pre-culture). **C,** Fluorescence microscopy of anti CA IX antibody binding to HT-29 and HeLa cells and flow cytometry quantification of anti-CA IX antibody binding to HT-29, HeLa, HCT-116, and MDA-MB-231cells incubated under normoxic and hypoxic cultures. Up-regulation of CA IX expression was shown in hypoxic HT-29 and HeLa cells by both fluorescence microscopy and flow cytometry quantification, confirming the ELISA results in HT-29 and HeLa cells. The flow cytometry quantification results of HT-29 and HeLa cells were compared with binding of antibodies to CA IX negative HCT-116 and MDA-MB-231 cells, showing a background binding of antibodies to HCT-116 and MDA-MB-231 cells under both hypoxic and normoxic cultures.
(TIF)

Figure S2 **Pharmacokinetic study of HS680.** The results show the plasma clearance profile of HS680 after an intravenous injection and the calculations of plasma half-lives in mice. The fast and slow half-lives of HS680 found to be 2 min and 2.67 h, respectively.
(TIF)

Figure S3 **Localizations of HS680 and tumor hypoxia biomarkers in HeLa xenografts and other tissues.** Collected tumor, kidney and muscle tissues were snap-frozen in OCT, sectioned (8 µm), and stained with anti CA IX antibody and anti-pimonodazole antibody. HS680 and control agent signal are represented in red. Anti-CA IX and pimonidazole staining (green) were used as positive controls for hypoxia. Hoechst staining (blue) was used to indicate regions of vascular perfusion. In tumor tissue sections, Anti-CA IX antibody, pimonidazole, and HS680 were localized in hypoxic regions. The hypoxia markers were not detected in the muscle tissue sections. Low levels of HS680 and control agent, but not anti-CA IX antibody and pimonidazole, were observed in the kidney cortex areas suggesting that the signal in kidney was non-mechanistic and might related to the kidney clearance of the agents.
(TIF)

Figure S4 **Localizations of HS680 and tumor hypoxia biomarkers in HT-29 xenograft tissues.** A, The tissue staining patterns of the control agent, HS680, and HS680+AZ (red) from the same or adjacent tumor sections as staining with fluorescent CA IX antibody or pimonidazole (green) and the Hoechst perfusion stain (blue). H&E staining of tissue sections that were used for localization are shown on the left side of images. HS680 was specifically localized in regions with low Hoechst staining indicative of low oxygen (less perfused) but positive staining with both the CA IX antibody and pimonidazole. Pre-injection of the mice with unlabeled AZ blocked the binding of HS680 to control levels. B, Co-localization (overlay) of HS680 with CA IX antibody or pimonidazole was shown on the right side images indicating HS680 was clearly co-localized with both anti-CA IX antibody and pimonidazole in the hypoxic regions of the tumor sections.
(TIF)

Protocol S1 **The detailed descriptions of materials and methods for validations of cellular hypoxia in vitro.** The protocol contains procedures for hypoxia biomarker pimonidazole (hypoxyprobe) binding assay, measurement of extracellular pH, quantification of CA IX protein in cell lysates, and detection and quantification of anti-CA IX antibody binding to hypoxic and normoxic cells.
(DOCX)
