# Supplementary figures and images for: In Vivo Imaging and Quantification of Carbonic Anhydrase IX Expression as an Endogenous Biomarker of Tumor Hypoxia
Source: PLoS One. 2012 Nov 30;7(11):e50860. doi: 10.1371/journal.pone.0050860 (PMC3511310; doi:10.1371/journal.pone.0050860)

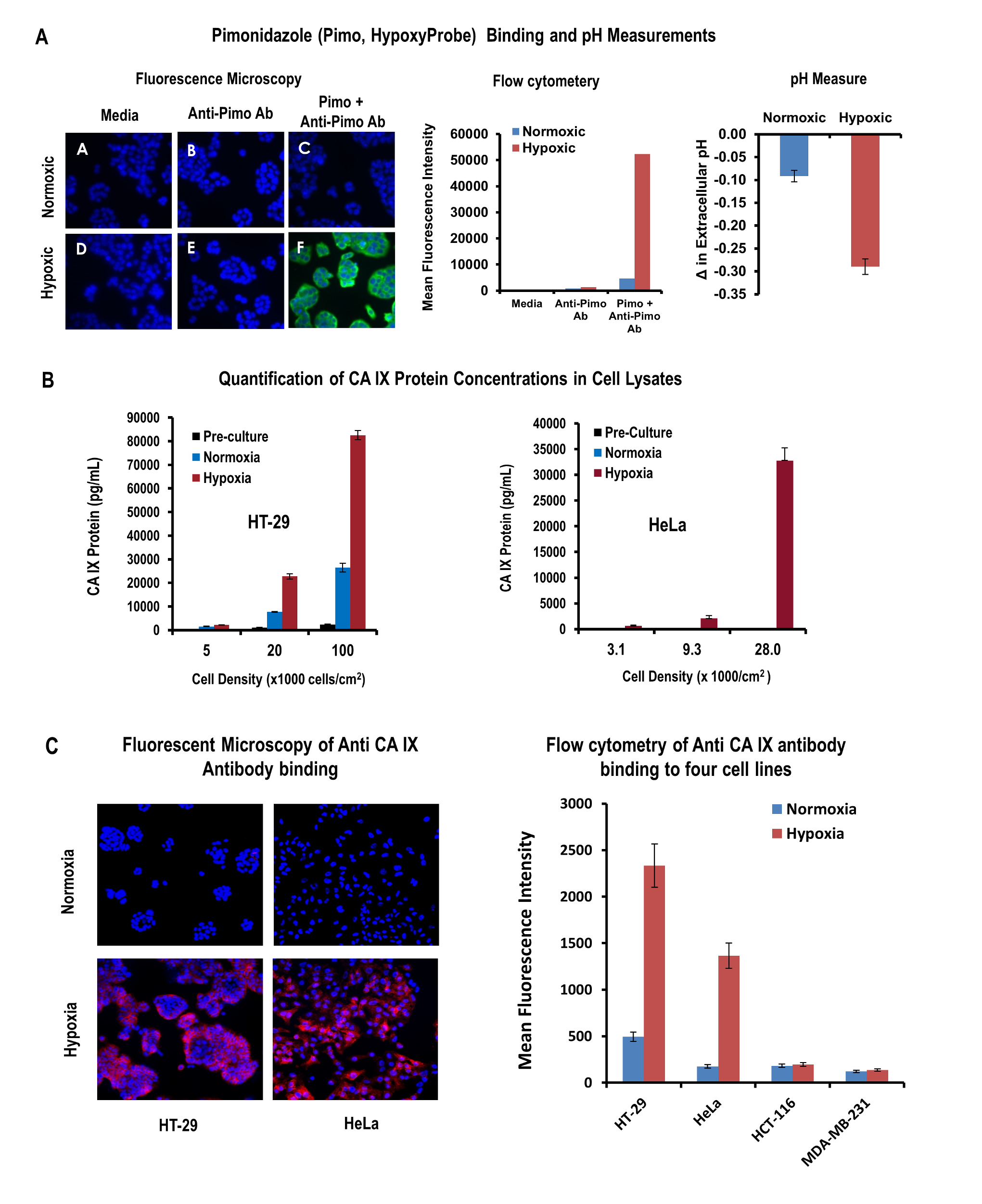

Supplement: Figure S1 — Validation of cellular hypoxia in vitro. A, Hypoxic induction in HT-29 cells was validated by pimonidazole (Pimo) binding using fluorescence microscopy and flow cytometry and by measuring culture media pH. HT-29 cells showed high levels of pimonidazole (exogenous hypoxia bio-marker) binding when cultured under hypoxic conditions, but not under normoxic conditions, very little signal was observed when the cells were incubated with FITC labeled anti-pimonidazole antibody (no pimonidazole) or with no agent (media) showing that pimonidazole binding was specific to hypoxic cells. The expected acidification associated with hypoxic cell culture conditions was confirmed by measuring the pH of the culture media. B, Quantification of CA IX protein levels in hypoxic and normoxic HT-29 and Hela cell lysates by CA IX ELISA. Effects of cell types and seeding cell densities are shown. CA IX protein was up-regulated in both cell types when cells were cultured in hypoxic condition, increasing 5 to 20 fold depending on the cell densities. An increased expression of CA IX was known when HT-29 cells cultured at higher densities in normoxic cultures. Under hypoxic cultures, CA IX expression in HT-29 cells was further increased 3 to 5 fold. In contrast, HeLa cells expressed very low levels CA IX protein in under normal oxygen conditions, regardless of cell density, with CA IX protein up-regulated greatly (>20-fold) in under low oxygen conditions. Little or no CA IX was detected in the cells prepared for the cultures (pre-culture). C, Fluorescence microscopy of anti CA IX antibody binding to HT-29 and HeLa cells and flow cytometry quantification of anti-CA IX antibody binding to HT-29, HeLa, HCT-116, and MDA-MB-231cells incubated under normoxic and hypoxic cultures. Up-regulation of CA IX expression was shown in hypoxic HT-29 and HeLa cells by both fluorescence microscopy and flow cytometry quantification, confirming the ELISA results in HT-29 and HeLa cells. The flow cytometry quantifica [file pone.0050860.s001.tif]

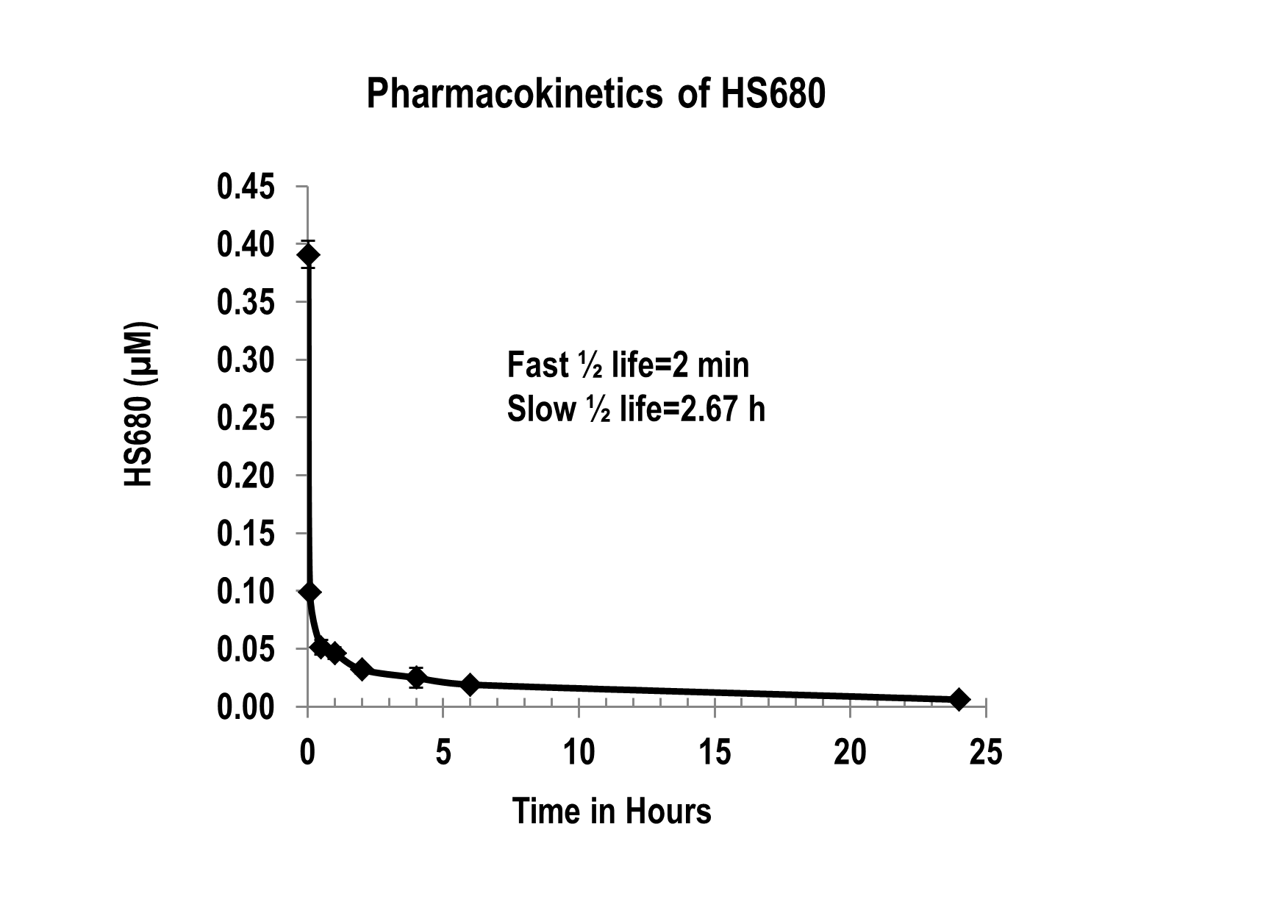

Supplement: Figure S2 — Pharmacokinetic study of HS680. The results show the plasma clearance profile of HS680 after an intravenous injection and the calculations of plasma half-lives in mice. The fast and slow half-lives of HS680 found to be 2 min and 2.67 h, respectively. (TIF) [file pone.0050860.s002.tif]

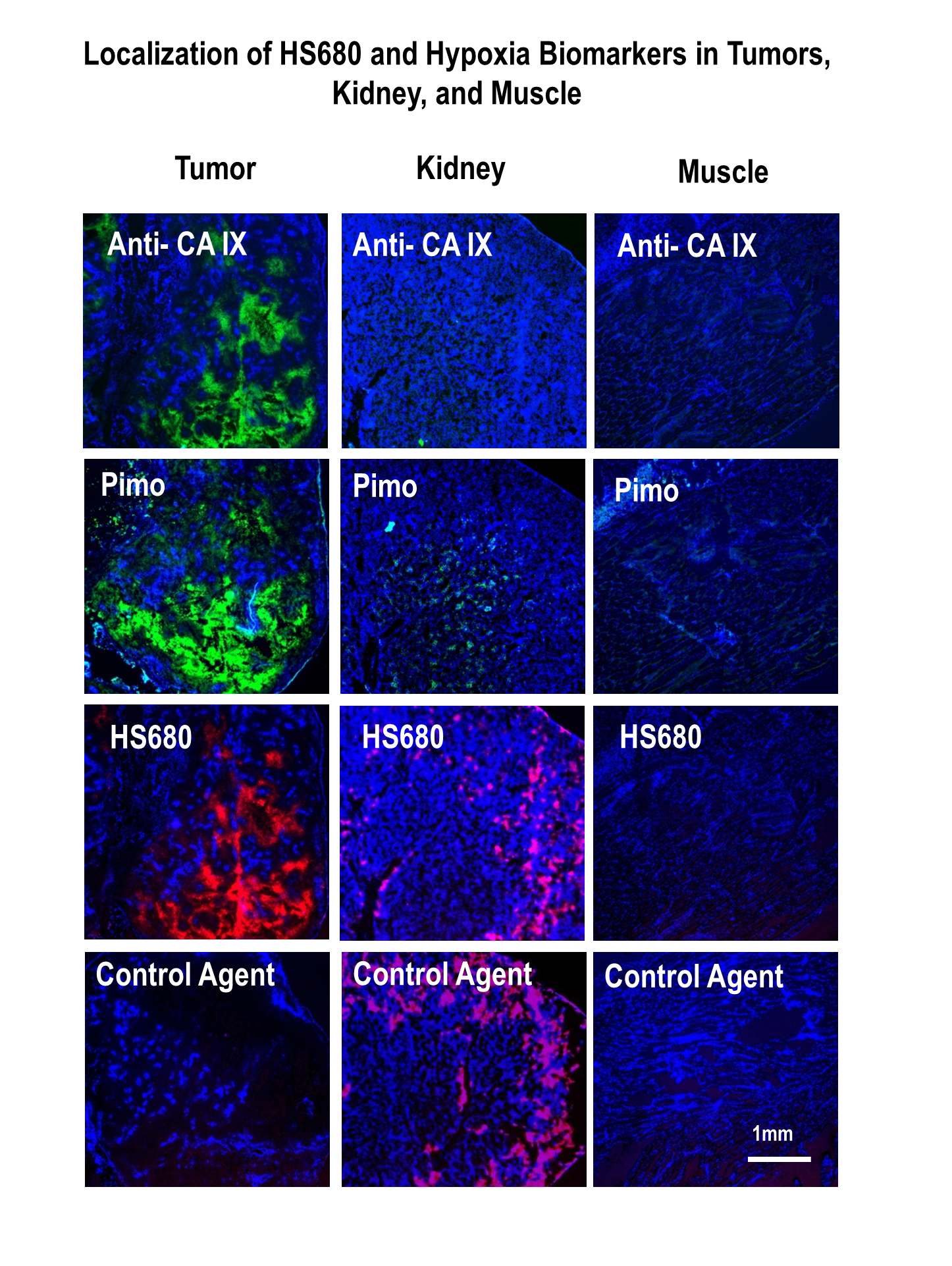

Supplement: Figure S3 — Localizations of HS680 and tumor hypoxia biomarkers in HeLa xenografts and other tissues. Collected tumor, kidney and muscle tissues were snap-frozen in OCT, sectioned (8 µm), and stained with anti CA IX antibody and anti-pimonodazole antibody. HS680 and control agent signal are represented in red. Anti-CA IX and pimonidazole staining (green) were used as positive controls for hypoxia. Hoechst staining (blue) was used to indicate regions of vascular perfusion. In tumor tissue sections, Anti-CA IX antibody, pimonidazole, and HS680 were localized in hypoxic regions. The hypoxia markers were not detected in the muscle tissue sections. Low levels of HS680 and control agent, but not anti-CA IX antibody and pimonidazole, were observed in the kidney cortex areas suggesting that the signal in kidney was non-mechanistic and might related to the kidney clearance of the agents. (TIF) [file pone.0050860.s003.tif]

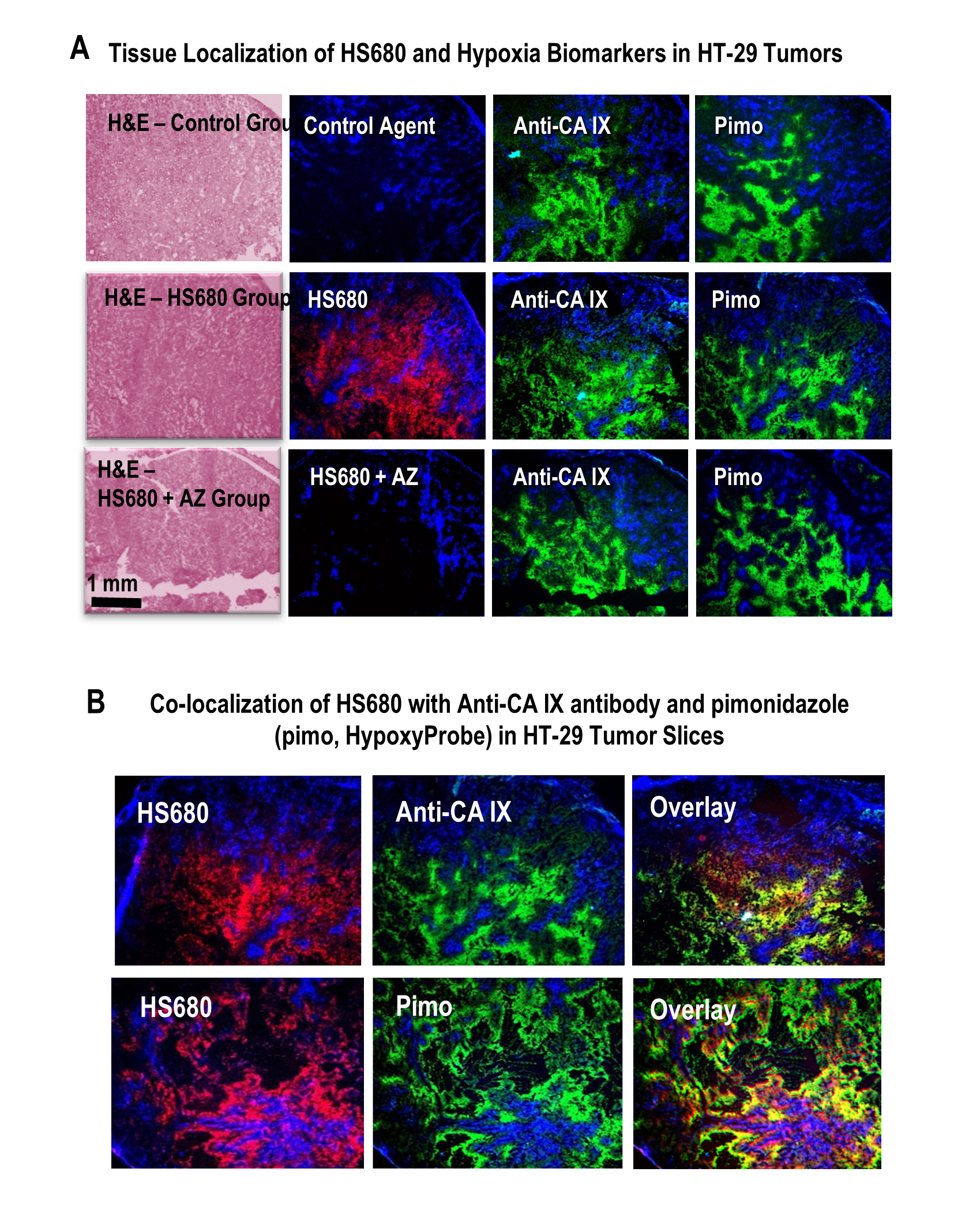

Supplement: Figure S4 — Localizations of HS680 and tumor hypoxia biomarkers in HT-29 xenograft tissues. A, The tissue staining patterns of the control agent, HS680, and HS680+AZ (red) from the same or adjacent tumor sections as staining with fluorescent CA IX antibody or pimonidazole (green) and the Hoechst perfusion stain (blue). H&E staining of tissue sections that were used for localization are shown on the left side of images. HS680 was specifically localized in regions with low Hoechst staining indicative of low oxygen (less perfused) but positive staining with both the CA IX antibody and pimonidazole. Pre-injection of the mice with unlabeled AZ blocked the binding of HS680 to control levels. B, Co-localization (overlay) of HS680 with CA IX antibody or pimonidazole was shown on the right side images indicating HS680 was clearly co-localized with both anti-CA IX antibody and pimonidazole in the hypoxic regions of the tumor sections. (TIF) [file pone.0050860.s004.tif]
