# Supplementary material for: In Vivo Imaging and Quantification of Carbonic Anhydrase IX Expression as an Endogenous Biomarker of Tumor Hypoxia
Source: PLoS One. 2012 Nov 30;7(11):e50860. doi: 10.1371/journal.pone.0050860 (PMC3511310; doi:10.1371/journal.pone.0050860)
Supplement: Protocol S1 — The detailed descriptions of materials and methods for validations of cellular hypoxia in vitro. The protocol contains procedures for hypoxia biomarker pimonidazole (hypoxyprobe) binding assay, measurement of extracellular pH, quantification of CA IX protein in cell lysates, and detection and quantification of anti-CA IX antibody binding to hypoxic and normoxic cells. (DOCX) [file pone.0050860.s005.docx]

**Protocol S1**

***Pimonidazole Binding.*** Briefly, 200 μM pimonidazole (Hypoxyprobe-1^TM^ plus kit, Hypoxyprobe Inc., Burlington, MA) was incubated with the hypoxic and normoxic cells during the last 2 hour cultures. The cells were then washed twice, fixed with 70% ETOH for 10 min, and permeabilized with PBS containing 0.1% Triton 100 and 4% Fetal Bovine Serum (FBS) for 10 min. FITC-conjugated murine anti-pimonidazole monoclonal antibody then was added (200 uL/well for slide chamber and 1 mL/well for 6-well plate) and incubated at 37°C for 2.5 hours to detect pimonidazole trapping. Cells were then prepared for fluorescence microscopy visualization and flow cytometry quantification of pimonidazole trapping.

***Measurement of Extracellular pH.*** Changes in culture media pH were assessed by using procedures published previously [[1-3](#_ENREF_1)]. In brief, the culture media were collected at the time of replacing the media with diluted antibodies or after completed incubation with test agents in flow cytometry assays. Care was taken to ensure that cultures grown in normoxia and hypoxia were subconfluent and contained similar cell numbers. pH of collected media was measured immediately by a digital pH meter.

***Quantification of CA IX Protein in Cell Lysates:*** Up-regulation of CA IX protein expression in hypoxic cell lysates was quantified by human CA IX ELISA assay kit (DCA900, R&D Systems, Minneapolis, MN) as described previously [[4-6](#_ENREF_4)]. Briefly, cells cultured in six-well culture plates under hypoxic and normoxic conditions were rinsed with cold-PBS and lysed with 0.5 mL/well cold-RIPA buffer with HALT Protease Inhibitors (Pierce, Rockland, IL). The cells lysates were then transferred to microfuge tubes and centrifuged at 14000 rpm for 15 minutes. The supernatant was collected and stored at -80ºC until the assay was performed. To measure CA IX protein levels within cell lysates, serially diluted samples and standards were added to microtiter plates coated with capture antibody. Subsequently, detection antibody and substrate solution were added. The colormetric density of all samples were measured by a microplate reader at 450 nm, and CA IX concentrations were calculated according to a standard curve as described by manufacture.

***Detection and Quantification of Anti-CA IX Antibody Binding to Hypoxic and Normoxic cells.*** Hypoxia induction and up-regulation of CA IX were validated with anti-CA IX antibodies in CA IX positive HT-29 and HeLa and negative HCT-116, and MDA-MB-231cells. Hypoxic and normoxic cells were incubated with anti-CA IX antibodies and the cells were then prepared for fluorescence microscopy and flow cytometry. For fluorescence microscopy, anti-human CA IX antibody (FAB2188A, R&D System) was added directly to the culture media (1:50 dilution) to prevent re-oxygenation of the hypoxic cells. Upon completion of 1 hour incubation, the media was discarded and cell wells were rinsed twice with cold PBS and removed from the slides. The slides were dried at room temperature for 4 to 5 minutes, and the nuclear staining reagent DAPI (Invitrogen) was added prior to mounting with a coverslip. The slides were examined under fluorescence microscopy at appropriate fluorescence wavelengths, and images were acquired using an equal exposure time and the same magnification for comparisons. For flow cytometry, anti-human CA IX antibody (FAB2188F, R&D System) was added directly to the cell wells without replacing the media (1:100 dilution). After 1 hour incubation, cell wells were rinsed with cold PBS and cells were scraped from the plates using a cell scraper with 2 mL of PBS. Cells were then transferred to 5 mL tubes and spun for 10 minutes at 1000 rpm. To each tube, 500 uL PBS was added to re-suspend the cells for flow cytometry.

**References**

1. Cecchi A, Hulikova A, Pastorek J, Pastorekova S, Scozzafava A, et al. (2005) Carbonic anhydrase inhibitors. Design of fluorescent sulfonamides as probes of tumor-associated carbonic anhydrase IX that inhibit isozyme IX-mediated acidification of hypoxic tumors. J Med Chem 48: 4834-4841.

2. Dubois L, Douma K, Supuran CT, Chiu RK, van Zandvoort MA, et al. (2007) Imaging the hypoxia surrogate marker CA IX requires expression and catalytic activity for binding fluorescent sulfonamide inhibitors. Radiother Oncol 83: 367-373.

3. Svastova E, Hulikova A, Rafajova M, Zat'ovicova M, Gibadulinova A, et al. (2004) Hypoxia activates the capacity of tumor-associated carbonic anhydrase IX to acidify extracellular pH. FEBS Lett 577: 439-445.

4. Said HM, Hagemann C, Staab A, Stojic J, Kuhnel S, et al. (2007) Expression patterns of the hypoxia-related genes osteopontin, CA9, erythropoietin, VEGF and HIF-1alpha in human glioma in vitro and in vivo. Radiother Oncol 83: 398-405.

5. Tanaka N, Kato H, Inose T, Kimura H, Faried A, et al. (2008) Expression of carbonic anhydrase 9, a potential intrinsic marker of hypoxia, is associated with poor prognosis in oesophageal squamous cell carcinoma. Br J Cancer 99: 1468-1475.

6. Zhou GX, Ireland J, Rayman P, Finke J, Zhou M (2010) Quantification of carbonic anhydrase IX expression in serum and tissue of renal cell carcinoma patients using enzyme-linked immunosorbent assay: prognostic and diagnostic potentials. Urology 75: 257-261.
